# Increasing the frequency of plant-based food intake in daily diets reduces the risk of cardiovascular disease among elderly Chinese: a cohort study

**DOI:** 10.3389/fnut.2024.1440025

**Published:** 2024-07-15

**Authors:** Xin-Zheng Hou, Qian Wu, Qian-Yu Lv, Ying-Tian Yang, Lan-Lan Li, Xue-Jiao Ye, Chen-Yan Yang, Yan-Fei Lv, Shi-Han Wang

**Affiliations:** ^1^Department of Cardiovascular Diseases, Guang'anmen Hospital, China Academy of Chinese Medical Sciences, Beijing, China; ^2^College of Management, Fudan University, Shanghai, China

**Keywords:** CLHLS, plant-based food, cardiovascular disease, elderly Chinese, risk

## Abstract

**Objective:**

There is limited research on the relationship between the frequency of plant-based food intake and the risk of cardiovascular disease (CVD) among elderly Chinese. This study aims to evaluate the association between plant-based dietary index (PDI) and CVD risks, providing evidence for elderly Chinese to reduce CVD risks by increasing the frequency of plant-based food consumption.

**Methods:**

This study analyzed data from the Chinese Longitudinal Healthy Longevity Survey (CLHLS) 2011–2018, employing a multivariate modified Poisson regression model, trend tests, and restricted cubic spline (RCS) analysis to assess the linear and non-linear relationship between the PDI and CVD risks. Subgroup analyses and interaction tests were conducted to evaluate the robustness and population-specificity of the results.

**Results:**

This study included a total of 1,414 elderly Chinese, and at the end of follow-up, 487 participants had developed CVD. The multivariate modified Poisson regression model revealed a negative association between PDI and CVD risks [RR = 0.983, 95%CI = (0.970, 0.997)]. Similarly, the multivariate trend test (*p* = 0.031) and RCS analysis (*P* for nonlinear = 0.600) indicated a linear relationship between PDI and CVD risks. Subgroup analyses showed that the relationship between PDI and CVD risk was not influenced by gender, BMI, smoking, alcohol use, or exercise.

**Conclusion:**

The PDI was negatively correlated with CVD risks, indicating that increasing the frequency of plant-based food intake in the diet may reduce CVD risks among elderly Chinese.

## Introduction

Population aging is a challenging area worldwide, and the cardiovascular health of the elderly population is a striking field (1, 2). Approximately 17.9 million people worldwide die from cardiovascular diseases (CVD) every year (3). In China, as of 2015, approximately 290 million people were suffering from CVD (4). CVD has put great pain and a heavy economic burden on elderly Chinese. In China, the disability adjusted life years caused by CVD have reached 58.2255 million, accounting for approximately 20% of global CVD and nearly 9% higher than the international average (5). The annual direct medical expenses for CVD in China exceeded 130 billion yuan, accounting for more than 22% of the total medical expenses during the same period (4). In recent years, risk factors such as obesity, elevated blood pressure, and dyslipidemia have become prevalent among the elderly Chinese, which has further led to an increase in the incidence and mortality of CVD (6, 7). The prevention of CVD in elderly Chinese still faces huge challenges, and relevant preventive measures need to be explored (2).

The role of the diet in CVD prevention has attracted much attention. For example, fresh fruits and vegetables, as easily obtainable and cost-effective plant-based foods, have been found to have a negative correlation with CVD risks. A meta-analysis involving 4 million people found that high intake of fruits and vegetables can reduce CVD risk by 7%, coronary heart disease risk by 12%, and stroke risk by 18% (8). And study conducted on elderly Chinese reached similar conclusions (9). Compared with research on a single type of plant diet, it seems to be more practical to include the most common daily foods in the evaluation of dietary patterns. A prospective cohort study conducted among 123,330 postmenopausal women found that adhering to a plant-based dietary pattern can reduce risks of CVD, coronary heart disease, and heart failure by 11, 14, and 17%, respectively (10). In the above study, the evaluation of the dietary pattern included multiple food types, such as plant protein, nuts, viscous fiber, phytosterols, and saturated fat/cholesterol sources. A protective effect of a plant-based dietary pattern, including multiple food types, on CVD has been found in young and middle-aged people (11). However, there is little research on the correlation between the plant-based dietary pattern and CVD among the elderly Chinese. Evidence on whether elderly Chinese can reduce the risk of CVD by Increasing the frequency of plant-based foods is insufficient. Therefore, this study explored the correlation between the plant-based dietary index (PDI), an index that takes into account the frequency of multiple food intake in the daily diet, and CVD risks based on a nationwide elderly cohort study conducted in China, providing evidence for the elderly Chinese to reduce CVD risks by increasing the frequency of plant-based dietary intake in the diet.

## Methods

### Study design and participants

Data in this study came from the Chinese Longitudinal Healthy Longevity Survey (CLHLS). CLHLS is a community-based national prospective cohort study (12). The survey was established in 1998 and randomly selected representative samples from 23 provinces in China. New member recruitment and follow-up interviews were conducted every 3–4 years. Trained interviewers conducted surveys in participants’ homes using structured questionnaires, and when participants were unable to answer questions, a proxy interviewer (usually a spouse or other close relative) answered. The questionnaire involved rich individual micro-data on the elderly’s demographics, physical and mental health, behavior, diet and nutrition, living habits, and socioeconomic status. More details about CLHLS have been reported in previous studies (13, 14). CLHLS conformed to the principles outlined in the Declaration of Helsinki and was approved by the Peking University Ethical Review Board (IRB00001052-13074). All participants voluntarily agreed to participate in this study and signed an informed consent form upon participation. CLHLS data has been opened to the academic community and society for free use through the Peking University Open Research Data Platform.^1^

Our study analyzed data from the 2011 wave of the CLHLS, and the follow-up survey was conducted in 2018. Based on the research purpose and previous studies (15), we restricted samples. The following samples were excluded: 1. Individuals with CVD at baseline; 2. Participants under the age of 65; 3. Individuals without plant-based diet index at baseline; 4. Participants lost or died during follow-up.

### Dietary pattern assessment

To assess dietary patterns, dietary intake frequencies at baseline were obtained through a dietary questionnaire survey. The evaluation method we used adapted from the method of Zhu et al. (16). We constructed the plant-based dietary index (PDI) based on the frequency of food intake. A simplified food frequency questionnaire was used to collect dietary frequency information. This questionnaire covered the most common foods in the Chinese diet. Plant foods included fresh fruits, fresh vegetables, vegetable oils, bean products, mushrooms, garlic, nuts, tea, sugar, and salt-preserved vegetables. Animal foods include animal fat, meat, fish, eggs, and dairy products.

We calculated PDI by scoring the frequency of food intake. The questionnaire marked the frequency of food intake as “almost every day,” “not every day, but at least once per week,” “not every week, but at least once per month,” “not every month, but occasionally,” and “rarely or never,” which was used for bean products, mushrooms, garlic, nuts, tea, sugar, salt-preserved vegetables, fish, eggs and dairy products. The intake frequency of fresh fruits and fresh vegetables was marked as “almost every day,” “quite often,” “occasionally,” and “rarely or never.” For plant foods, we scored 5 for the most frequent intake, and with the gradual decrease in intake frequency, the score was reduced by 1 in turn. Instead, we scored 5 as the least frequent animal food intake and 1 as the most frequent animal food intake. As for cooking oil, vegetable oil scored 5 and animal fat scored 1. The PDI was obtained by summing the scores of all food categories. The PDI theoretically ranged from 14 to 70. The higher the participants’ PDI, the more inclined they were to the plant-based dietary pattern. Detailed evaluation criteria for the PDI can be found in the supplementary document. Previous studies have demonstrated that it is feasible and reliable to use food intake frequency rather than food intake to assess dietary patterns (17, 18). For example, when evaluating fruit and vegetable intake levels, frequency is more important than quantity (19).

### Outcomes ascertainment

The outcome of this study was self-reported nonfatal CVD, which mainly includes hypertension, heart disease, and stroke. Outcomes were obtained from participants’ health status surveys at follow-up in 2018. Survey questions were “Do you suffer from hypertension?,” “Do you suffer from heart disease?,” and “Do you suffer from a stroke?” If the participant answered “yes” to any of the above questions, it was defined as having CVD. Participants who answered “no” to all of the above questions were defined as not having CVD. The reliability of CLHLS health status data has been demonstrated in other studies (9).

### Covariates

Covariates, including demographic characteristics and health behaviors, were collected at baseline. Sociodemographic data included age, gender, spouse (whether having a spouse), co-residence (whether living alone), education, financial status (whether financial support met daily expenses), and annual physical examinations (whether annual physical examinations were routinely performed). Health behaviors included smoking status, alcohol consumption, and daily exercise. Participants were asked “Do you smoke at present?” and “Did you smoke in the past?” We defined current smokers as those who smoked at the interview, former smokers as those who smoked in the past but not at the interview, and non-smokers as those who neither smoked in the past nor at the interview. Similar evaluation methods were used for alcohol consumption and daily exercise. In addition, the body mass index (BMI) was also collected, which was calculated from the height and weight of the participants.

### Statistical analysis

Participants were divided into two groups according to outcomes to describe the characteristics of study population. Continuous variables were expressed as mean and standard deviation or median and quartile, and the *t*-test or Kruskal-Wallis rank sum test was selected for hypothesis testing according to applicable conditions. Classified variables were expressed as absolute numbers and percentages, and the chi-square test was used for hypothesis testing.

The relationship between PDI and CVD risks was assessed using multivariate Poisson regression with robust variance estimation (20). PDI was included in the model, respectively, as continuous variables, two-category variables (with the median as the cut-off point), and four-category variables (with the quartiles as the cut-off point). To control confounding bias, we constructed three regression models by including different potential confounding factors. No confounding factors were included in Model 1. Model 2 included demographic factors, including age and gender. Model 3 further incorporated BMI, smoking status, alcohol consumption, and daily exercise based on Model 2. BMI was converted into a three-category variable when included in the model (with 18.5 and 23.9 as cut points). We also conducted trend tests for the four categories of PDI. The variance inflation factor was used to evaluate multicollinearity between variables. If it was greater than 5, variables were excluded. The multivariate restricted cubic spline (RCS) was used to evaluate the dose–response relationship between PDI (continuous variable) and CVD risks. To explore whether the results were population-specific, we conducted stratified analyses according to gender, BMI, smoking status, alcohol consumption, and daily exercise. The product terms of these variables and PDI were included, respectively, in the model to evaluate the interaction effects.

Data analysis was completed by software IBM SPSS Statistics 26.0 (IBM Corp., Armonk, N.Y., USA) and R 4.3.0 (R Foundation for Statistical Computing, Vienna, Austria). *p* < 0.05 on both sides was considered statistically significant.

## Results

### Sample characteristics

**Figure 1:**
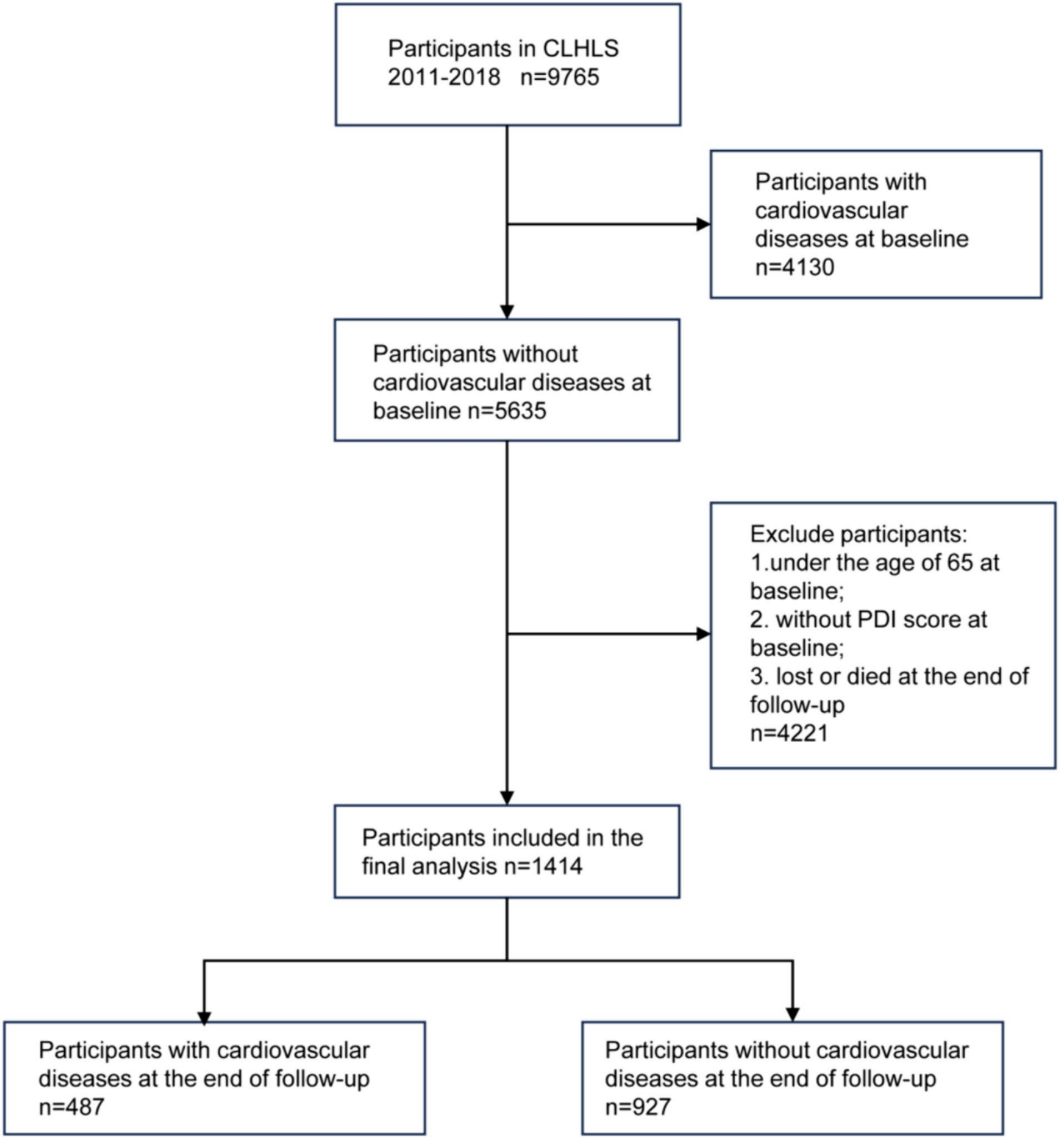
Study population screening flow chart.

The study population screening process was shown in Figure 1. A total of 9,765 participants took part in the CLHLS 2011–2018. After screening according to inclusion and exclusion criteria, 1,414 participants, with complete data on PDI and CVD, were included in our study. Study population characteristics were shown in Table 1. The average age of the participants was 78.13 ± 8.74. The mean PDI was 40.21 ± 5.79, which indicated that most participants followed a mixed plant-based and animal-based eating pattern. A total of 487 (34%) participants had developed CVD at follow-up in 2018. They were younger and had a higher BMI than those without CVD. The univariate analysis revealed no statistically significant differences in terms of PDI, gender, education, smoking, alcohol consumption, and physical exercise among participants with different CVD outcomes.

**Table 1:** Baseline characteristics of the study population.

Variables	Total participants (*n* = 1,414)	Without CVD (*n* = 927)	With CVD (*n* = 487)	*p* value
Age, years, Mean ± SD	78.13 ± 8.74	78.71 ± 9.09	77.03 ± 7.93	<0.001
BMI, Kg/m^2,Mean ± SD	21.66 ± 3.79	21.36 ± 3.68	22.24 ± 3.95	<0.001
PDI, Mean ± SD	40.21 ± 5.79	40.34 ± 5.71	39.98 ± 5.93	0.276
Gender, *n* (%)				0.205
Male	721 (50.99)	484 (52.21)	237 (48.67)	
Female	693 (49.01)	443 (47.79)	250 (51.33)	
Co-resident, *n* (%)				0.232
Living alone	267 (19.04)	183 (19.96)	84 (17.32)	
Not living alone	1,135 (80.96)	734 (80.04)	401 (82.68)	
Spouse, *n* (%)				0.035
Without spouse	591 (41.97)	406 (43.99)	185 (38.14)	
Having a spouse	817 (58.03)	517 (56.01)	300 (61.86)	
Education, *n* (%)				0.841
Sixth grade below	1,169 (82.85)	765 (82.70)	404 (83.13)	
Sixth grade and above	242 (17.15)	160 (17.30)	82 (16.87)	
Financial support, *n* (%)				0.947
Sufficient	1,120 (79.49)	734 (79.44)	386 (79.59)	
Insufficient	289 (20.51)	190 (20.56)	99 (20.41)	
Annual physical examination, *n* (%)				0.198
Yes	434 (30.87)	274 (29.72)	160 (33.06)	
No	972 (69.13)	648 (70.28)	324 (66.94)	
Smoking status, *n* (%)				0.288
Current smoker	326 (23.22)	225 (24.46)	101 (20.87)	
Former smoker	184 (13.11)	116 (12.61)	68 (14.05)	
Never smoker	894 (63.68)	579 (62.93)	315 (65.08)	
Alcohol consumption, *n* (%)				0.600
Current drinker	320 (22.94)	208 (22.71)	112 (23.38)	
Former drinker	160 (11.47)	100 (10.92)	60 (12.53)	
Never drinker	915 (65.59)	608 (66.38)	307 (64.09)	
Daily exercise, *n* (%)				0.636
Long-term exerciser	530 (37.97)	350 (38.21)	180 (37.50)	
Former exerciser	98 (7.02)	60 (6.55)	38 (7.92)	
Never exerciser	768 (55.01)	506 (55.24)	262 (54.58)	

BMI, body mass index; PDI, Plant-based dietary index.

### The relationship between PDI and CVD risks

**Figure 2:**
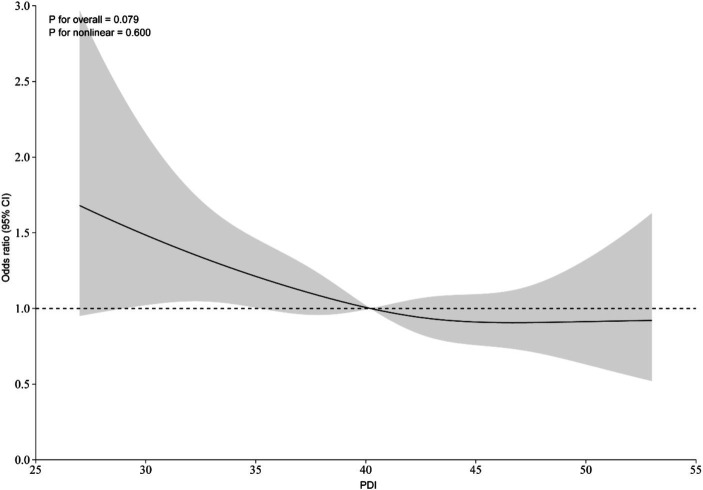
Restricted cubic spline plot of PDI and CVD risk. The solid line represents the risk ratio, and the gray shading represents the 95% confidence interval of the risk ratio.

The relationship between PDI and CVD risks was shown in Table 2. Univariate modified Poisson regression found no correlation between PDI and CVD risks [RR = 0.993, 95%CI = (0.981, 1.006)]. After adjusting for potential confounding factors, including age, gender, BMI, smoking status, alcohol consumption, and daily exercise, multivariate modified Poisson regression showed a negative correlation between PDI and CVD risks [RR = 0.983, 95%CI = (0.970, 0.997)]. For every 1 increase in PDI, CVD risks decreased by 1.7%. Compared with participants in the lowest PDI score interval (Q1), participants in the high PDI score interval (Q2, Q3, Q4) had a 22.8, 18.4, and 22.0% reduction in CVD risks, respectively. The trend test after adjusting for potential confounding factors showed that the linear trend between PDI and CVD risks was statistically significant (*p* = 0.031). The multivariate RCS more intuitively indicated that CVD risks had a negative correlation with PDI, and the linear trend was statistically significant (*P* for nonlinear = 0.600), which were shown in Figure 2.

**Table 2:** The relationship between PDI and CVD.

	Model 1	Model 2	Model 3
PDI (Continuous variable)	0.993 (0.981,1.006)	0.988 (0.976,1.001)	0.983 (0.970,0.997)
PDI (Binary variable)			
< Median	1	1	1
≥ Median	0.984 (0.852,1.137)	0.945 (0.817,1.092)	0.909 (0.781,1.057)
PDI (Quadripartite variable)			
Q1	1	1	1
Q2	0.815 (0.666,0.996)	0.774 (0.633,0.946)	0.772 (0.628,0.948)
Q3	0.883 (0.726,1.075)	0.829 (0.681,1.009)	0.816 (0.667,0.997)
Q4	0.907 (0.746,1.103)	0.845 (0.693,1.029)	0.780 (0.632,0.963)
*P* for trend	0.398	0.124	0.031

Model 1: No covariates were adjusted. Model 2: Age and gender were adjusted. Model 3: Age, gender, BMI, smoking status, alcohol consumption, and daily exercise were adjusted.

### Subgroup analysis

**Figure 3:**
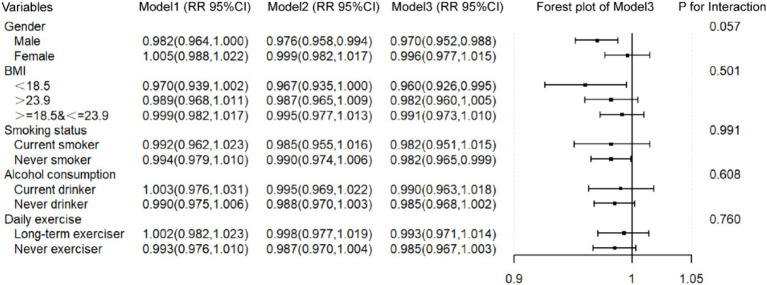
The relationship between PDI and CVD in subgroup populations. Model 1: No covariates were adjusted. Model 2: Age and gender were adjusted. Model 3: Age, gender, BMI, smoking status, alcohol consumption, and daily exercise were adjusted. When a variable was used as a stratification variable, it was not adjusted.

To examine the robustness and population-specificity of results, we conducted subgroup analyses and interaction tests on variables potentially related to CVD risks, including gender, BMI, smoking, alcohol consumption, and physical exercise. In Model 3, the inverse association between PDI and CVD risks remained consistent across subgroups defined by gender, BMI, smoking, alcohol consumption, and physical exercise, although some effect sizes did not reach statistical significance, likely due to the reduced sample size in subgroups. Specifically, the inverse association between PDI and CVD risks remained statistically significant in male [RR = 0.970, 95%CI = (0.952, 0.988)], low BMI participants [RR = 0.960, 95%CI = (0.926, 0.995)], and never smokers [RR = 0.982, 95%CI = (0.965, 0.999)]. Furthermore, no interactions were observed between PDI and any of the stratifying variables. The results of the subgroup analyses and interaction tests were presented in Figure 3.

## Discussion

**Figure 4:**
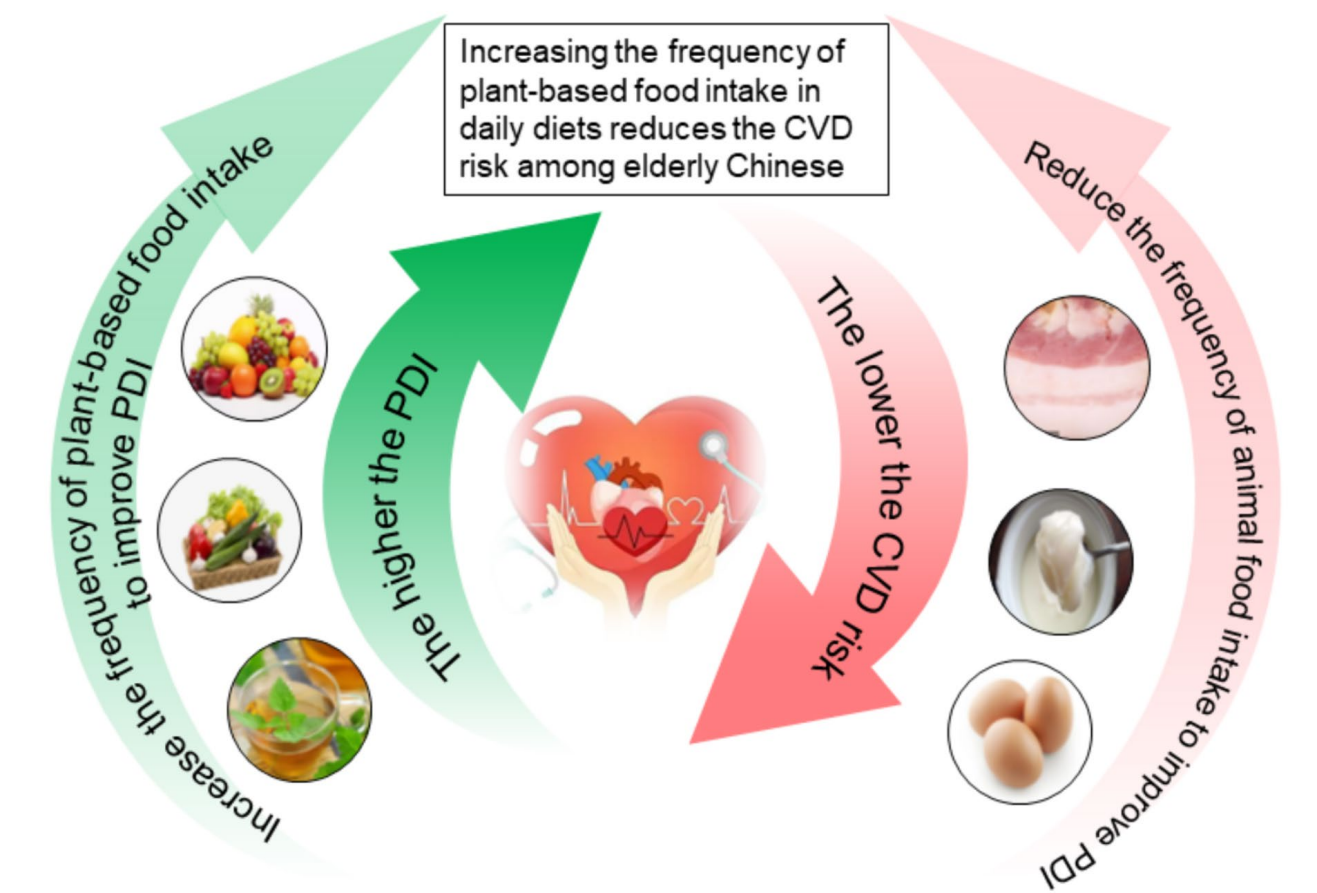
The relationship between PDI and CVD risk in subgroup populations. Model 1: No covariates were adjusted. Model 2: Age and gender were adjusted. Model 3: Age, gender, BMI, smoking status, alcohol consumption, and daily exercise were adjusted. When a variable was used as a stratification variable, it was not adjusted.

This study conducted a retrospective cohort study based on the CLHLS data. Using multivariable modified Poisson regression and RCS, we evaluated the association between PDI and CVD risks, aiming to provide evidence for reducing CVD risks among elderly Chinese through increased frequency of plant-based food intake. We found a negative correlation between PDI and CVD risks after controlling for confounding bias through multivariable modified Poisson regression. For every 1 increase in PDI, the CVD risk decreased by 1.7%. PDI in the highest quartile reduced CVD risk by 22.0% compared to the lowest quartile. Both the trend test and the RCS showed that PDI had a linear correlation with CVD. Subgroup analysis has yielded similar results, indicating that our results should be robust. Increasing the frequency of plant-based food intake among elderly Chinese could reduce the CVD risk, as shown in Figure 4.

The protective effect of plant-based dietary patterns on CVD in the real world may be greater than our estimation. In our study, the average age of participants was 78.13 ± 8.74 years old, which is roughly equivalent to the life expectancy of Chinese people (21). Therefore, our research subjects are more inclined toward the long-lived population. The average BMI was 21.66 ± 3.79, which was within a reasonable range. The proportions of current smokers and current drinkers were 23.22 and 22.94%, respectively. Compared with other CVD studies on elderly Chinese, our study population had fewer CVD risk factors (22–24). However, other studies have found that a plant-based diet has a significant control effect on many cardiovascular risk factors, such as blood sugar, blood lipids, blood pressure, body weight, and so on (25–27). Therefore, plant-based dietary patterns may further reduce the risk of CVD by controlling CVD risk factors in the real world.

This study achieved similar results to some previous studies. A cohort study conducted among postmenopausal women in the United States found that adhering to a plant-based diet can reduce the risk of coronary heart disease, heart failure, and overall CVD (10). However, the above-mentioned relationship was not found in our study among Chinese elderly women, which may be caused by the age difference of study populations. A prospective cohort study in Spain found that a healthy plant-based dietary pattern including fruits, vegetables, nuts, legumes, vegetable oils, and tea/coffee reduced the risk of CVD death by 37% (28). In addition, a plant-based diet has been found to control atherosclerosis-related high blood pressure and urinary microalbumin (25, 27). Most of the above-mentioned studies were conducted in Europe and America, and studies conducted in Asia showed similar results. A cohort study (29) conducted among middle-aged Koreans classified dietary patterns into healthy plant-based dietary patterns and unhealthy plant-based dietary patterns based on the types of plant foods. After controlling for confounding bias, no correlation was found between the healthy plant-based dietary pattern and the risk of CVD mortality. This suggests that more studies on the relationship between plant-based dietary patterns and CVD risks need to be conducted to clarify the relationship. The research on plant-based foods and CVD risks conducted in China currently mostly focuses on a single type of food. For example, a study has found that increasing the frequency of fresh fruit intake among middle-aged and elderly Chinese can reduce the risk of coronary artery events by 34% (30). In addition, studies have found that consuming high-quality plant-based foods such as fresh vegetables, nuts, and tea has cardiovascular protective effects on elderly Chinese (9, 31, 32). Compared to studies that focused on a single type of food, it seems more realistic to consider the variety of daily foods comprehensively. Previous study (16) on the cognitive function of elderly Chinese have comprehensively evaluated the plant-based dietary pattern. We used a similar study method and found a negative correlation between the plant-based dietary pattern and CVD risks in elderly Chinese, which was consistent with the conclusions of most studies.

This study provides some evidence for the formulation of public health policies related to the elderly Chinese. Increasing clinical practice guidelines are beginning to include dietary guidance, and approximately half of the guidelines recommend a plant-based dietary pattern (33). However, previous studies on the elderly diet focused more on protein intake and recommended increasing protein intake in the elderly to prevent age-related loss of muscle strength and function (34). There are also studies indicating that increasing plasma albumin content can reduce the CVD risks (35). Previous studies on the relationship between plant-based dietary patterns and CVD risks have mostly been conducted in Western populations, while studies on the elderly Chinese mostly focused on individual food types. Our study method included most types of daily food for the elderly Chinese. Our study further supports public health and clinical recommendations that increasing the frequency of plant-based food intake in the elderly Chinese is beneficial for preventing the occurrence of CVD.

Based on the nationally representative CLHLS cohort, this study explored the relationship between PDI and CVD risks, providing evidence for reducing the risk of CVD among the elderly Chinese by increasing the frequency of plant-based food intake. However, there are some limitations in this study. Firstly, the sample population comprises elderly individuals, thus the conclusion applies to the elderly Chinese, and further study is needed to determine whether it applies to other populations. Secondly, the CLHLS dietary questionnaire only investigated the frequency of food intake, without investigating the intake quantity or nutrient content of each food. In future studies, information such as food intake frequency, quantity, and nutrient content can be considered comprehensively. Additionally, while the CLHLS implemented quality control measures in multiple processes such as research design and implementation, the potential for information bias in questionnaire surveys still needs to be vigilant. Finally, this study did not collect laboratory test data, and future studies can include auxiliary diagnostic indicators to further control confounding biases.

## Data availability statement

The original contributions presented in the study are included in the article/supplementary material, further inquiries can be directed to the corresponding author.

## Ethics statement

Ethical approval was not required for the studies involving humans because the study used publicly available data.

## Author contributions

X-ZH: Writing – original draft, Writing – review & editing. QW: Writing – review & editing. Q-YL: Writing – review & editing. Y-TY: Writing – review & editing. L-LL: Writing – review & editing. X-JY: Writing – review & editing. C-YY: Writing – review & editing. Y-FL: Writing – review & editing. S-HW: Writing – review & editing.

## Footnotes

^1^ http://opendata.pku.edu.cn/
